# Invisible but Impactful: Understanding the Dynamics and Effects of Gender Microaggressions Through Workers’ Eyes

**DOI:** 10.3390/bs16101864

**Published:** 2026-10-09

**Authors:** Francesco D’Anna, Martina Pansini, Ilaria Buonomo, Paula Benevene

**Affiliations:** 1 Department of Human Sciences, LUMSA University, 00193 Rome, Italy; i.buonomo1@lumsa.it (I.B.); p.benevene@lumsa.it (P.B.); 2 Department of Theoretical and Applied Sciences, Research Centre for Applied Psychology, e-Campus University, 22060 Novedrate, Italy; martina.pansini@uniecampus.it

**Keywords:** gender microaggressions, social sustainability, diversity, equity, and inclusion (DEI), workplace inclusion, gender inequality, employee well-being, power asymmetries

## Abstract

Subtle and often ambiguous forms of gender-based bias continue to shape everyday workplace interactions, influencing how individuals are perceived, evaluated, and treated. This study examines gender microaggressions (GMAs), defined as subtle, often ambiguous verbal, behavioral, or environmental expressions of gender-based bias. These pervasive yet frequently overlooked dynamics are explored in terms of their directionality, multi-level effects, and how individuals interpret and respond to them within organizational hierarchies. Using a qualitative design, 47 semi-structured interviews were conducted with Italian workers across roles and sectors. Data were analyzed through thematic analysis, complemented by T-Lab co-occurrence analysis to identify recurring patterns in perceived effects and reactions. Participants predominantly perceived GMAs as targeting women and being enacted by men, reflecting perceived gendered power asymmetries. Participants reported cascading negative effects, including psychological distress, reduced motivation, impaired workplace relationships, and perceived effects extending to broader organizational functioning. Reactions were highly context-dependent, with non-response emerging as common due to ambiguity and power constraints, while participants also described more assertive responses following repeated experiences. Overall, GMAs emerge as systemic, multi-level phenomena that undermine individual well-being and organizational functioning, highlighting the need for structural and cultural interventions to foster more inclusive workplaces.

## 1. Introduction

In contemporary organizational contexts, sustainability has evolved beyond environmental concerns to encompass the social conditions that enable individuals and organizations to thrive over time. From a social sustainability perspective, workplaces are expected to foster equity, inclusion, well-being, and fair treatment (Colantonio, 2009; Kramar, 2014). Within this framework, both overt and subtle discrimination can be understood as a threat to social sustainability.

This study focuses on microaggressions, commonly defined as brief and often ambiguous everyday indignities that convey derogatory or exclusionary messages toward marginalized groups (Sue & Spanierman, 2020; Sue et al., 2007). Although individually subtle, these behaviors accumulate over time, producing significant detrimental consequences (Costa et al., 2022). Their implicit and often deniable nature makes them particularly difficult to identify and address, positioning microaggressions as pervasive yet largely invisible threats to inclusive and socially sustainable organizational environments.

Microaggressions can therefore be understood as involving two related dimensions: (a) the observable act or behavior through which they are expressed and (b) the harmful message that the act conveys to the target. Although the literature generally agrees that microaggressions may be enacted intentionally or unintentionally (Sue et al., 2007), it has not yet reached full consensus on the relative role of these two dimensions in determining whether an interaction should be classified as a microaggression (Lilienfeld, 2017; Mekawi & Todd, 2021; Williams, 2020). In particular, it remains debated whether an interaction must be perceived as offensive or harmful by the target to constitute a microaggression, or whether an act may be classified as microaggressive even when its recipient does not consciously perceive or experience it as such. This unresolved issue is particularly relevant for observational research, which requires sufficiently clear criteria for determining which observed behaviors should be classified as microaggressive.

Within this broader framework, gender microaggressions (GMAs) represent a specific form of bias grounded in socially constructed norms and expectations about gender. GMAs include subtle comments, behaviors, or environmental cues that reinforce gender hierarchies, question competence on the basis of gender, or invalidate individuals’ gender identities and experiences (Sue & Spanierman, 2020; Nadal, 2018). While research on microaggressions has often drawn on models developed in the study of racial bias, gender-based dynamics introduce distinct patterns shaped by gender roles and power asymmetries (Sue & Spanierman, 2020).

Importantly, GMAs are conceptually related to, yet distinct from, sexual harassment. Both phenomena stem from gender inequality and contribute to the reproduction of gendered power structures in organizations (Lopez et al., 2009; Sue & Spanierman, 2020; Wilson & Thompson, 2001). However, whereas sexual harassment typically involves unwanted sexualized behaviors or explicit gender-based hostility that meet legal or policy-based definitions (Fitzgerald, 1990), GMAs more often take the form of subtle, ambiguous, and sometimes unintentional acts (Sue & Spanierman, 2020). In practice, the boundaries between the two may blur, as some behaviors can simultaneously function as microaggressions and as normalized or low-intensity forms of harassment (Sue & Spanierman, 2020). At the same time, not all forms of sexual harassment are “micro” (Wilson & Thompson, 2001), and not all GMAs entail sexualized or legally defined harassment (Sue & Spanierman, 2020). Recognizing both the overlap and the distinction between these constructs is essential to fully capture the spectrum of gender-based mistreatment in the workplace.

### 1.1. Gender Microaggressions in the Workplace: Dynamics and Gaps in the Literature

Building on the conceptualization of GMAs as subtle yet structurally embedded forms of gender bias, the workplace represents a central arena in which these dynamics are enacted and sustained. Organizational contexts are structured by hierarchies, role expectations, evaluation systems, and informal norms that shape everyday interactions and reproduce gendered power relations (Cortina, 2008). Within these environments, seemingly minor exchanges may function as mechanisms through which competence, authority, and belonging are differentially attributed.

In professional settings, GMAs can take the form of interruptions, differential standards of competence, role confinement, exclusion from informal networks, or subtle questioning of authority. Because such behaviors are often normalized within organizational culture, they may be difficult to identify and challenge. Moreover, their enactment and interpretation are shaped by hierarchical position, gender composition, and contextual norms, suggesting that directionality and impact are relational rather than fixed.

Although literature on microaggressions is growing (Costa et al., 2022; Smith & Griffiths, 2022), key gaps remain. Limited attention has been paid to the perceived directionality of GMAs across gender and power configurations, and microaggressions are often treated as a homogeneous construct despite evidence that different forms may produce distinct consequences (Costa et al., 2022). Moreover, some European contexts, particularly Italy, remain underexamined.

To address these gaps, this study aims to advance the literature by examining how Italian workers perceive and interpret (a) the gendered directionality of GMAs in everyday interactions, (b) their personal and professional consequences, and (c) how workers make sense of and respond to GMAs within organizational hierarchies.

### 1.2. Research Questions

#### 1.2.1. Gendered Directionality of GMAs

Understanding the gendered directionality of GMAs, namely who is perceived as targeting whom, is important for understanding the dynamics underlying this phenomenon. Existing literature suggests that men and women may perceive and interpret GMAs differently (Basford et al., 2014), reflecting divergent understandings of inclusion, fairness, and well-being (Colantonio, 2009). However, this literature may also shape expectations about the directionality of GMAs. Previous research has often approached GMAs through gendered assumptions about their directionality. In particular, GMAs have predominantly been examined as behaviors directed toward women (Algner & Lorenz, 2022), and some studies have focused exclusively on women’s experiences. While this perspective has contributed to our understanding of gender-based mistreatment, it may also limit the range of perceptions considered.

Limited attention has been paid to how workers themselves perceive the directionality of GMAs when they are given the opportunity to describe these dynamics openly. An open approach can help capture how workers spontaneously identify who targets whom, without assuming a priori which gender is more likely to enact or experience GMAs. This perspective may provide a broader understanding of how gendered power relations are perceived and constructed in everyday organizational interactions. Accordingly, the following research question is proposed:

RQ1: How do workers perceive the gendered directionality of GMAs in the workplace, in terms of who is perceived as targeting whom?

#### 1.2.2. Multilevel Effects of GMAs

Prior scholarship has also shown that microaggressions tend to produce negative consequences for individuals, including diminished well-being and psychological distress (Costa et al., 2022), while discriminatory workplace behaviors more broadly have been associated with detrimental organizational outcomes (Smith & Griffiths, 2022).

Although research on GMAs has grown in recent years, little is known about how workers themselves perceive and articulate these effects across different domains (Smith & Griffiths, 2022). Exploring consequences at multiple levels allows for a more nuanced understanding of how GMAs are experienced and interpreted in practice. To examine these dynamics, the following research question is proposed:

RQ2: What effects do workers attribute to GMAs at the personal, work-related, and organizational levels?

#### 1.2.3. Reactions to GMAs

Given the ambiguity that often characterizes microaggressions, individuals may interpret and respond to them in diverse ways, with implications for the maintenance of inclusive and socially sustainable work environments. While prior research has acknowledged coping strategies and resistance behaviors (Lewis et al., 2013; Sanchez et al., 2018), less attention has been devoted to how workers make sense of their reactions within organizational power structures.

Building on the multilevel impact of GMAs outlined above, it is particularly relevant to examine how employees describe their responses to unsustainable acts, how they assess the appropriateness or risks of reacting, and how power relations and contextual constraints shape these choices (Sue & Spanierman, 2020). Understanding these processes is essential to capture how everyday interactions may either reproduce or challenge the conditions that sustain equity and inclusivity in organizations. Accordingly, the following research question is formulated:

RQ3: How do workers describe and make sense of their reactions to GMAs in the workplace?

## 2. Materials and Methods

### 2.1. Participants and Data Collection

This study employed one-on-one semi-structured interviews with a sample of Italian workers. This approach was selected because the study aimed to explore how workers spontaneously perceive, interpret, and respond to GMAs, including the meanings, effects, and reactions they associate with these experiences, rather than to establish their objective occurrence or prevalence. Given the subtle nature of GMAs, interviews allow participants to describe in their own terms which interactions they perceive as gendered microaggressions and how they interpret and respond to them (Sue & Spanierman, 2020).

Eligible participants were Italian men and women with at least one year of work experience. Prior to the interviews, participants provided basic demographic information. A snowball sampling technique was adopted, whereby each participant was asked to refer additional potential participants.

The interviews were conducted by two trained interviewers, one male and one female. Both interviewers were cisgender and presented a gender expression consistent with their identified gender, without exaggerated traits. They were instructed to maintain strict neutrality throughout each interaction, and interviews in which neutrality was compromised beyond an acceptable threshold were deemed invalid for analytical purposes. Both interviewers were under 31 years old (male: 27; female: 29). To reduce potential interviewer effects, interviews were initially distributed approximately equally across the two interviewers. However, interviewer availability toward the end of data collection resulted in an uneven distribution that could not be fully rebalanced. Given this imbalance, the possibility that interviewer gender influenced participants’ responses cannot be excluded, and differences across interviewer-gender groups were therefore interpreted cautiously. Similarly, given the relatively young age of both interviewers, the possibility that age differences between interviewers and older participants may have influenced participants’ comfort or responses cannot be excluded.

Out of 95 individuals initially contacted, 54 agreed to participate, yielding 54 interviews. One was excluded because the participant did not meet the minimum work-experience requirement. An additional six interviews were excluded due to deviations from the standardized interviewing protocol that could compromise interviewer neutrality, including leading or gender-suggestive questioning (3) and overly affective interviewer–participant exchanges (3). These exclusions were jointly assessed by the research team based on the interview recordings and predefined neutrality criteria. Consequently, the final dataset consisted of 47 valid interviews. All interviews were conducted in Italian.

Table 1 summarizes the sample distribution with respect to the interviewees’ self-reported gender, the interviewers’ gender, and the age group to which each interviewee belongs.

Regarding occupational roles, 11 participants held top-level managerial positions, 7 were in mid-level management, and 30 occupied non-managerial roles. The total number of reported roles exceeds the number of participants, as one participant reported holding two distinct roles across different sectors. In addition, 13 participants worked in the healthcare sector, making it the most represented occupational sector in the sample. Table 2 provides a breakdown of the occupational sectors represented in the sample. As some participants reported holding multiple roles across different sectors, the frequencies reported in Table 2 exceed the total number of participants.

To protect participants’ privacy and minimize the risk of workplace identification, detailed questions about specific workplace characteristics were avoided. This supported more open accounts but limited the possibility of systematic sector-specific comparisons, except for healthcare, which was sufficiently represented and thematically consistent to be analyzed separately.

Notably, all participants identified as cisgender. As such, this article uses the terms ‘man’ and ‘male’ and ‘woman’ and ‘female’ interchangeably. The experiences of transgender or non-binary individuals fall outside the scope of this research and are not addressed here. Of the 47 interviews, 4 were conducted in person and the rest remotely.

Participants were anonymized using randomly generated identification codes, applied post-interview, with only the research team able to link codes to personal identities. For ease of reading, the codes were structured to align with Table 1. Each code consists of three letters and a number (e.g., MMO1): the first letter indicates the interviewer’s gender (M = male, F = female), the second the interviewee’s gender (M = male, F = female), and the third the interviewee’s age group (U = under 31, X = 31–50, O = over 50). The final number distinguishes individuals within the same category (e.g., MMO1 and MMO2).

The study was conducted in accordance with the Declaration of Helsinki and was approved by the Ethics Committee of LUMSA University (No. 5/2024) on 8 April 2024. Informed consent was obtained from all participants prior to participation, including consent to data use and audio recording.

### 2.2. Interview Characteristics

At the start of each interview, the researcher explained the session structure, data protection measures, and ethical considerations. Participants were then asked to provide informed consent for participation, data usage, and audio recording.

Interviews were conducted in person, by phone, or via videoconferencing tools, according to participants’ preferences. They were open-ended in duration, and participants were encouraged to respond as they wished. Participants were informed that they could answer freely, indicate that they did not know or did not remember, or decline to answer any question. They were also reminded that they could withdraw from the interview at any time.

A three-phase, semi-structured format allowed interviewers to adapt the conversation to individual experiences.

The first section was focused on workers’ spontaneous perceptions of GMAs. To capture participants’ initial understanding of GMAs without priming, the conversation began with broad, exploratory questions (“What comes to mind when you think of gender microaggressions in the workplace?”, “Are there any particular dynamics that come to mind?”, “Have you ever witnessed, experienced, or heard about such events?”). This section aimed to elicit perceptions unfiltered by the interviewer’s framework or terminology.

Importantly, participants were invited to report whether they had encountered or noticed GMAs without being forced to classify the experience as personally experienced, enacted, directly observed, or reported by others. This non-directive approach was intended to avoid assigning participants to predefined target or perpetrator roles and to allow them to describe, in their own terms, the workplace experiences they associated with GMAs. Consequently, the accounts included personal, observed, and vicarious experiences, although the specific perspective from which some accounts originated was not always explicitly identifiable.

After participants had shared their initial, unprompted views, the interviewer introduced a brief, orally adapted version of Sue and colleagues’ (Sue & Spanierman, 2020; Sue et al., 2007) definition of GMAs. Participants were presented with the simplified formulation: “Subtle verbal or non-verbal indignities, whether intentional or unintentional, directed at an individual or group, that promote gender-based discrimination in the workplace”. Following this, the interviewer also provided a short overview of the three categories of GMAs proposed by Sue and Spanierman (2020) to support subsequent discussion. This phase provided a common conceptual framework for the subsequent discussion, while allowing the initial spontaneous accounts to be considered in relation to established definitions of GMAs.

In the final part, participants were asked to discuss any perceived consequences at the personal, workgroup, or organizational level. Before closing the interview, participants were invited to share any further observations they considered pertinent.

Throughout the interview, no predefined response options were provided, and participants could indicate that they perceived no impact, did not know, or did not recall any consequences related to GMAs. To preserve the open-ended and non-directive nature of the interviews, participants were not asked specific questions about the characteristics of their workplace or immediate work context (e.g., gender distribution). This approach served both to protect participants’ privacy and to avoid directing their accounts toward particular workplace dynamics or gender-related contextual factors through potentially leading questions.

### 2.3. Data Analysis

All interviews were manually transcribed and analysed using both manual thematic analysis and T-Lab software (version 10). In the manual phase, researchers closely read all transcripts, identified recurring themes, and categorized relevant excerpts accordingly.

The T-Lab analysis was conducted to further explore underlying patterns within the domains of effects and reactions emerging from participants’ narratives, complementing the qualitative thematic findings. Accordingly, T-Lab supported the identification of key constructs and thematic patterns through automated processing. All 47 full interview transcripts (in Italian) were uploaded and lemmatized, with irrelevant or ambiguous terms removed. The software’s default settings were retained throughout the analysis (minimum frequency = 5; co-occurrence threshold = 3; *p* < 0.05). Semantically related words were grouped, yielding 354 lemmas. A co-occurrence analysis was then conducted, producing the final co-occurrence graphs based on statistically significant associations. Relevant items were subsequently translated into English to ensure clarity.

### 2.4. Qualitative Coding Procedure

The interview transcripts were analyzed using a thematic coding approach (Braun & Clarke, 2006). One researcher conducted an iterative process of reading and re-reading the transcripts to identify and categorize participants’ experiences, perceptions, and reported effects and reactions related to workplace gender microaggressions. Coding continued until no substantively new categories emerged, with the aim of capturing the full range of participants’ accounts. The resulting coding framework was subsequently reviewed by the research team, which monitored the process and discussed and approved the final set of codes and their thematic organization.

This process resulted in 99 codes relating to the effects of GMAs and 11 codes relating to participants’ reactions. Conceptually related codes were subsequently consolidated into broader thematic domains, resulting in the personal, work-related, and organizational effects presented in the Results section. While the Results section focuses on the most salient findings, the complete coding framework is provided in Supplementary Material S1.

## 3. Results

The qualitative analysis identified several interconnected patterns concerning the characterization, effects, and reactions associated with workplace gender microaggressions (GMAs). To facilitate the interpretation of the findings, Table 3 provides an overview of the main thematic domains and key themes emerging from the analysis. The subsequent sections examine each domain in greater detail, while the T-Lab co-occurrence analyses provide complementary and illustrative representations of selected patterns.

### 3.1. Characterization of GMAs in the Workplace

At the beginning of the interview, participants were invited to freely describe gender microaggressions (GMAs) in the workplace. GMAs were predominantly framed as behaviors targeting women, who were consistently identified as the primary targets by all 47 participants. Nevertheless, 26 participants also acknowledged that men may experience GMAs, although, as expressed by MFX8, “always to a lesser extent” than women.

In addition to women and men, 13 respondents identified other groups as vulnerable to GMAs, including transgender individuals. More particularly, MMU2 defined GMAs as “an experience that tends to disadvantage women or, more generally, someone who occupies a position different from that of a heterosexual cisgender man.”

Interestingly, 9 participants referred to homosexuality as a characteristic associated with exposure to GMAs, despite its conceptual distinction from gender (Fausto-Sterling, 2019).

Another relevant finding concerns the perceived directionality of GMAs. A total of 38 respondents explicitly referred to microaggressive behaviors enacted by men toward women. In contrast, only 11 participants described instances of microaggressions from women toward men. Finally, participants also reported same-gender microaggressions. Nineteen respondents described GMAs occurring between women, whereas 9 participants referred to microaggressions occurring between men.

Table 4 synthesizes these distributional patterns, showing a marked predominance of women as perceived targets and of men-to-women GMAs in participants’ accounts.

Regarding perceived frequency, participants’ accounts further reinforced the predominance of women as the primary targets (15) and men as the primary perpetrators (10) of GMAs.

This perceived man-to-woman pattern was linked by 8 participants to entrenched power dynamics affecting women in workplace contexts, which MFX5 described as “atavistic.” In addition, FFU2 and FFO2 attributed men’s enactment of GMAs to a tendency toward dominance, particularly in male-dominated fields.

Interestingly, 6 female participants described microaggressions between women as particularly severe, although less frequent than those enacted by men. FFU2 suggested that “women themselves, especially with other women, also tend to put twice as much aggression into it. It is as if […] sometimes women also internalize a stereotype.” Similarly, FFO1 emphasized the targeted nature of such behaviors: “Women toward women, they are terribly mean […] They know exactly where to hit you to hurt you […] women are masters at this.”

Lastly, 17 participants explicitly referred to workplace GMAs arising from gender-based differential treatment. Of these, 16 participants reported that such differential treatment systematically advantaged men. Women were described as being subjected to unrealistic job expectations, aggressive and humiliating communication styles, unequal reward systems, disparaging verbal comments, restricted career opportunities, and a lack of trust in their professional competence.

In contrast, only FMX3 described experiences in which he perceived gendered differences in treatment that favored female colleagues. He reported being assigned more physically demanding or stressful tasks because he was a man, while also experiencing harsher verbal treatment: “I had to take on certain jobs or tasks simply because I was a man, […] perhaps jobs that were a bit more physically demanding, or that considerably increased the level of stress. […] And because I was a man, they could, so to speak, insult me. […] With a girl, they wouldn’t have done that.”

### 3.2. Effects of GMAs in the Workplace

Analysis of the interviews identified 99 distinct codes related to the effects of GMAs, which were subsequently grouped into the main thematic domains of effects (Braun & Clarke, 2006), as anticipated in Table 3. While this paper focuses on the most relevant findings, the complete coding framework is provided in Supplementary Material S1.

Before examining personal, work-related, and organizational effects separately, two cross-domain patterns emerged across these three levels: the context-dependent nature of GMA effects and their cumulative, cascading development.

Participants emphasized that the impact of GMAs cannot be understood as uniform or static. Twenty-three respondents reported that GMA effects vary depending on several factors, including the characteristics of the target, the specific context in which the microaggression occurs, the type of GMA, and the broader work environment.

Moreover, 15 participants highlighted the interrelated nature of these effects, describing them as unfolding through a harmful chain reaction that often originates at the individual level and progressively extends to interpersonal and group dynamics, eventually affecting the organization as a whole. As MFO4 linked this to top-down GMAs causing employees to stop working properly: “If the microaggression is from the top […] the people at the bottom, who are the ones who do, let’s put it this way, the dirty work […] if you start not doing [your job], or doing it badly, […] then the whole company is affected.”

Figure 1 offers an illustrative preview of the patterns discussed in greater detail in the subsequent sections, where personal, work-related, and organizational effects are presented.

### 3.3. Personal Effects of GMAs in the Workplace

Focusing on the personal dimension of impact (effects shaping individuals’ lived experiences), the analysis identified 41 codes related to personal-level effects. Nearly all respondents reported detrimental consequences of GMAs on personal well-being, with only 3 participants not referring to any personal-level effects.

Overall, 33 participants referred to emotional consequences in their accounts. Participants described a wide range of negative emotional reactions, most notably frustration, fear, anger, and anxiety.

Beyond emotional reactions, 26 respondents reported additional mental health consequences associated with GMAs. The most frequently cited outcomes were threats to individual serenity (8), psychological distress (8), and depressive symptoms (5). MFO2 described these experiences as a prolonged “tragic mental loop,” underscoring their cumulative and self-perpetuating nature over time. Similarly, MFX1 reported “a feeling of […] helplessness, that nothing will ever change,” suggesting how repeated exposure may reinforce negative psychological states and the internalization of gendered stereotypes. In a more severe case, the psychological toll of workplace GMAs culminated in acute mental breakdowns that led MFU1 to quit her job.

Losses in self-esteem and self-confidence were reported by 28 participants, explicitly attributing these declines to experiences of microaggressions. For 15 respondents, GMAs were also associated with growing insecurities that shaped both professional and personal self-perceptions (Figure 1). MFU4 described how GMAs may undermine self-confidence, noting that “you are making that person lose confidence in themselves, creating insecurities.” Similarly, 8 participants described processes of self-doubt and self-conviction of “not being enough,” highlighting the internalization of stereotypes and humiliations to the point of reproducing them in their own self-evaluations.

Participants’ accounts extended beyond the psychological domain, also encompassing somatic and behavioral consequences (7), including inhibition of vital functions, changes in appetite, and increased reliance on substances as coping mechanisms. MMO3 described an elevated dependence on caffeine to maintain daily functioning. Additionally, 12 respondents reported difficulties in maintaining work–life balance, frequently describing the transfer of the burden from the workplace into private life, consistent with the spillover effect described in the literature (Staines, 1980).

Finally, although effects were mostly associated with negative outcomes (Figure 1), 4 respondents identified potential positive outcomes associated with exposure to GMAs. Participants framed microaggressions as experiences that, while harmful, contributed to the development of resilience, enhanced self-esteem, and a heightened sense of responsibility. MFU1 described perceiving GMAs as having “both a positive and a negative” impact, noting that “in a positive sense, it pushes you to do certain things, and that is okay.”

### 3.4. Work-Related Effects of GMAs in the Workplace

From a work-related perspective, the analysis identified 45 codes related to the detrimental effects of workplace GMAs on daily functioning and interpersonal dynamics.

Specifically, 46 participants mentioned a pervasive sense of workplace discomfort, manifesting in various forms detailed in this section. Among female participants, this discomfort was associated with perceptions of inadequacy regarding their role (7), as well as with a sense of powerlessness accompanied by thoughts of giving up (3). Furthermore, 3 respondents reported that GMAs might cause a persistent feeling of having to do more in order to meet expectations. Twelve participants emphasized that this pervasive discomfort contributes to an uneasy workplace climate. Such environments were described as draining employees’ energy and disrupting group harmony.

Motivational concerns also emerged as a salient theme, being addressed by 16 participants. In particular, GMAs were described as progressively eroding engagement and proactivity at work. Several respondents portrayed these experiences as undermining their willingness to remain actively involved, also reporting feelings of resignation and a diminished sense of agency. Some participants also linked GMAs to reduced organizational commitment and a weakened sense of attachment to their workplace.

Furthermore, interpersonal relationships were identified as particularly affected. Thirty participants reported negative effects on workplace relationships, particularly on team cooperation (19). These effects were attributed to the accumulation of interpersonal conflicts, erosion of group cohesion, and communication breakdowns. Some participants further linked these dynamics to reputational effects within the group, whereby GMAs may undermine the reputation and perceived standing of both targets and perpetrators.

Building on this, 23 participants reported that GMAs foster dynamics of devaluation within the workplace. These dynamics were primarily expressed through being ignored or belittled (12) and experiencing a lack of respect (5). Drawing on both direct and vicarious experiences, MFX8 described how women’s professional competence may be undermined through gendered assumptions and disparaging treatment: “[Patients] prefer male staff […] My professionalism as an operator is not recognized because I am a woman. [A patient] also insulted the doctor, saying that she ‘didn’t know shit,’ in his exact words […] completely denigrating the professional because she was a woman.” Women are frequently not recognized as individuals but rather treated as subordinates, and their opinions are often dismissed or overridden. Additionally, 12 participants noted that repeated GMAs foster workplace behaviors and attitudes that progressively isolate the victim. MMO2 and FFO5 interpreted this process as contributing to the suppression of diversity within the workplace.

Restrictions on freedom of expression also emerged as a recurrent theme. Eighteen participants reported that GMAs inhibit open expression in the workplace. Accounts emphasized the need to endure mistreatment tacitly to avoid backlash. In this regard, FFU3 described that “the emotional distress does not allow you, for example, to speak in public, to be more creative, to put forward your own ideas on a particular project, or to express your opinion more openly.”

Overall, defensive responses were frequently reported (15), while a smaller number referred to aggressive reactions (2). Submissive behaviors were also described by 9 participants, with no substantial gender differences in personal accounts. Notably, in healthcare settings, MFX9 reported submissive behaviors, such as “opening doors for physicians,” whereas MFX8 described patients’ continuous expectations of submission, both attributed to their role as nurses. FMU1 attributed this passivity to commitment to the profession and the need to maintain job security.

According to participants, discomfort resulting from GMAs may also have broader occupational consequences. Specifically, respondents reported an increased likelihood of leaving their job (11), encountering obstacles to career advancement (8), and experiencing general differences in treatment (12), mostly favoring men.

Lastly, 27 participants indicated that GMAs may threaten work quality and performance by impairing responsibility, objectivity, work organization, professionalism, and mental clarity, ultimately increasing the likelihood of task-related errors.

### 3.5. Organizational Effects of GMAs in the Workplace

From a broader organizational perspective, the analysis identified 11 codes capturing the potentially detrimental effects of workplace GMAs on organizational functioning. As noted by FMO3, these effects were framed as operating through a pyramidal process, whereby negative dynamics originating at lower levels progressively affect the organization as a whole.

Organizational culture emerged as a key area affected by GMAs. Three participants described the development of a toxic organizational culture resulting from repeated microaggressions. Similarly, FFO1 and FFO3 referred to cultural stagnation linked to persistent workplace aggressions. Cultural deterioration was also described as interfering with talent development and retention: four older participants noted that such environments may hinder the expression of individual talent, leading to a loss of valuable human capital. In addition, MMO2 referred to losses in what he defined as “organizational capital,” particularly through reduced knowledge exchange among workers that would otherwise contribute to organizational development.

In addition, 4 participants highlighted negative effects on organizational reputation and image. For instance, MFX5 referred to a loss of organizational credibility due to informal word-of-mouth: “you naturally end up telling your friends, relatives, or other people about your experience, and this also affects the credibility of the company, the workplace, or the organization, because it can also have an impact on its image when this kind of situation becomes known.”

Building on this, FFU2 further linked negative word-of-mouth to turnover and recruitment issues: “word gets around and it becomes difficult to find new people to join. […] This team has consisted of two people for about three years because a colleague left three years ago and they have never managed to find a replacement.”

### 3.6. Reactions to GMAs in the Workplace

Similar to the analysis of effects, the interviews yielded 12 distinct codes related to reactions, which were subsequently grouped into overarching themes and presented in this section.

Focusing on respondents’ personal and vicarious experiences with GMAs in the workplace, it is notable that immediate reactions of victims are highly variable. Across the sample, 42 participants recounted personal experiences of GMAs. Interestingly, 29 of them reported that in certain situations, victims did not react directly. For 6 participants, the initial response to the aggression was one of shock and disorientation, which prevented them from identifying an immediate or effective way to respond. Commonly, individuals simply withdrew from the situation, feeling discomfort, disgust, or intimidation, uncertain of how to act.

Non-reaction may also be related to a perceived lack of agency in responding to the situation. Indeed, MFX2 asserted: “Many women, when these things happen, do not respond. Most do not respond because, in the end, everything is brushed off with a little smile and a dismissive attitude.” Furthermore, MFO3 described how, in authoritarian work environments, victims silently express indignation while maintaining a façade of normalcy, concealing substantial underlying tension.

Non-reaction was further interpreted as a factor that may exacerbate negative responses: MFU1 suggested that passive responses increase vulnerability to stress and burnout over time, while MMU1 highlighted that some individuals carry the emotional burden home, venting privately. FFO1 contextualized non-reaction by noting that the lack of explicit provocations leaves victims feeling uncertain. Notably, 4 participants reflected that they would now respond more assertively if faced with the same situations.

Reactions were also widely understood to depend on context, individual characteristics, and situational factors. Twelve participants emphasized that the perceived severity of the threat and the victim’s personal resources can influence the intensity of the response. For instance, MMX1 observed that colleagues from lower socio-economic backgrounds tended to struggle more in reacting, whereas individuals from more privileged backgrounds often responded more assertively.

Moreover, 23 participants reported having actively reacted. Responses varied in form, with some going way beyond verbal interventions. FFO4, for example, described how, after repeated unwanted physical contact from her supervisor, she eventually reacted by moving her chair and physically pushing him away: “After four or five times, I reacted by moving the chair and simply hitting him where it hurts most […] and he never came close to me again.”

Others responded more subtly: for instance, MFX6 described seeking to defuse tension while maintaining professionalism and composure, pointing out the problematic situation without escalating it.

Interestingly, respondents also described delayed reactions, emerging only after repeated exposure to multiple microaggressions and reaching a point at which passive tolerance was no longer possible. For instance, MFX2 described how repeated exposure eventually led her to strong reactions: “It became more evident because I was more tired, but [the aggressor] did it more than once; it was just that time that I reacted particularly badly… at a certain point, one, two, three, four [times], and then I respond badly.”

Figure 2 illustrates these findings, highlighting the discomfort experienced when facing workplace microaggressions, especially those related to women. The analysis also shows strong co-occurrence patterns linking submissive responses to mistreatment and difficulties in asserting oneself, complementing the qualitative accounts.

## 4. Discussion

Taken together, the findings provide a direct account of how workers recognize, interpret, and respond to GMAs in the workplace, offering a broad spectrum of perceptions regarding their targets, directionality, consequences, and reactions.

Rather than portraying GMAs as uniform interpersonal events, participants’ accounts show them as gendered and cumulative processes whose meaning and consequences depend on how workers perceive and interpret the situation, as well as on the resources and context available to them. The findings therefore suggest that understanding workplace GMAs requires attention not only to the behaviors themselves, but also to how they are experienced and negotiated by those exposed to them.

### 4.1. Conceptualization, Targets, and Directionality of GMAs

Overall, the findings answer RQ1 by showing a relatively coherent perception of GMAs as gendered behaviors predominantly enacted by men toward women in participants’ accounts. These patterns should be understood as perceived gendered directionality rather than as independently verified differences between perpetrators and targets, consistent with the exploratory and non-directive design of the study.

This gendered pattern is consistent with the conceptualization of microaggressions as everyday denigrating exchanges directed toward individuals on the basis of group membership, with women representing a particularly salient target of gender-based microaggressions (Nelson & Olson, 2023; Sue & Spanierman, 2020). The strong predominance of women as perceived targets, together with the pronounced directionality from men toward women, is also consistent with research documenting the persistence of gender stereotypes and status-based disadvantage in organizational contexts (Cameron & Kulick, 2003; Eagly & Karau, 2002; Heilman, 2012). Importantly, however, participants also recognized men as potential targets, although these experiences were consistently perceived as less frequent and less severe.

This latter finding extends rather than contradicts the conventional understanding of GMAs. While the literature has predominantly examined microaggressions directed toward women and other marginalized groups (Smith & Griffiths, 2022), participants’ accounts indicate that gendered expectations may also constrain men, although through different normative pressures. The experience described by FMX3, for example, involved expectations that men should accept physically demanding tasks and tolerate harsher interpersonal treatment because they were men. Such accounts are consistent with research showing that masculinity is itself regulated through social expectations and that violations or challenges to masculine norms can generate gender-related threat and strain (Boettcher et al., 2019). Workplace research similarly conceptualizes organizational environments as sites in which men may be subject to pressures to demonstrate conformity to masculine norms, with consequences for both men and women (Berdahl et al., 2018). Thus, the present findings suggest that gender microaggressions may be directed not only downward along established gender hierarchies, but also toward individuals who are perceived as failing to conform to gendered expectations, including members of the socially dominant gender.

Participants’ recognition of transgender individuals and other gender minorities as particularly exposed to GMAs further underscores the role of normative gender expectations in shaping microaggressive dynamics (Marchi et al., 2024). Building on this, the inclusion of homosexuality as a perceived risk factor, despite its conceptual distinction from gender (Fausto-Sterling, 2019), can be interpreted in two complementary ways. First, it reflects a limited awareness of the distinction between gender identity and sexual orientation. In this sense, a person’s identity and attractions tend to be inaccurately collapsed into a single category (National Academies of Sciences, Engineering, and Medicine, 2022). Second, this pattern points to the broader regulatory function of GMAs, which operate to sanction deviations from hegemonic gender norms. Within this framework, gay men may be implicitly perceived as “less masculine,” while lesbian women may be stereotyped as masculine or “tomboyish,” in line with widely shared cultural assumptions (Kite & Deaux, 1987; Madon, 1997). This interpretation aligns with intersectional feminist approaches: GMAs function as mechanisms of gender norm enforcement, targeting not only women but also individuals whose identities or expressions are perceived as insufficiently aligned with dominant, cisgender, and heteronormative ideals (Kite & Deaux, 1987; Marchi et al., 2024; Schilt & Westbrook, 2009; Sterzing et al., 2017).

Rather than treating these categories as independent, an intersectional perspective therefore helps explain why the same gendered norm may be experienced differently depending on the target’s identity and perception, reinforcing the inherently subjective nature of microaggressions (Sue & Spanierman, 2020).

The occurrence of same-gender GMAs—especially woman-to-woman microaggressions—adds a further dimension to this pattern. Although less frequently reported than male-to-female GMAs, participants sometimes perceived these interactions as particularly severe and linked them to the internalization and reproduction of gender stereotypes. This finding is compatible with research showing that gender norms can be reproduced within rather than only across gender groups, highlighting that gendered disadvantage is not sustained exclusively through direct male-to-female interactions (Evteeva et al., 2024). In this sense, the present findings suggest that the directionality of GMAs is more complex than a simple perpetrator–victim dichotomy.

Taken together, these findings reflect participants’ perceived attribution of gendered roles. Participants’ accounts broadly support the centrality of women’s experiences while also suggesting that a comprehensive conceptualization of workplace GMAs should remain sensitive to variation in both target and mechanism. This distinction is particularly relevant for measurement: existing instruments have largely been developed to assess microaggressions against women in the workplace, such as the MIMI-16 (Algner & Lorenz, 2022). Extending such measures to men, however, should not simply involve replacing “women” with a generic gender label, as gendered expectations and forms of devaluation may differ across groups. Instead, measures intended to capture GMAs across the workforce should retain gender-specific content while allowing for different manifestations across target groups. The present findings therefore point toward a broader, yet still gender-sensitive, conceptualization of workplace GMAs.

### 4.2. Personal, Work-Related, and Organizational Effects of GMAs in the Workplace

The findings provide a broad map of the consequences attributed to GMAs, with 99 codes spanning personal, work-related, and organizational domains. Rather than pointing to a single outcome, this breadth suggests that, from workers’ perspectives, GMAs are experienced as interconnected processes whose consequences may extend beyond the immediate interpersonal encounter. This breadth is particularly relevant to the exploratory aim of the study, as it captures the range of ways in which workers recognize and make sense of GMA-related effects, including less visible consequences. The complete coding framework is provided in Supplementary Material S1.

A particularly salient finding is the perceived cascading nature of these effects. Participants described how an apparently localized interpersonal event may progressively affect relationships, team functioning, and, ultimately, the wider organization. MFO4’s description of a “pyramidal” process illustrates this perceived escalation, whereby negative dynamics affecting workers may progressively be transmitted to broader organizational functioning. This interpretation is consistent with conceptualizations of microaggressions as cumulative rather than isolated experiences, in which repeated exposure produces harm through accumulation and normalization—the so-called “death by a thousand cuts” (Andersson & Pearson, 1999; Sue & Spanierman, 2020; Sue et al., 2007). Importantly, participants’ accounts suggest that this accumulation may be perceived not only within individuals, but also across levels of workplace functioning, from personal experience to interpersonal and organizational dynamics.

At the personal level, the findings largely converge with previous research documenting the psychological burden associated with gendered mistreatment (Costa et al., 2022; Smith & Griffiths, 2022). Participants described a wide range of emotional difficulties, alongside somatic and behavioral consequences and spillover into private life (Pearson & Porath, 2005; Staines, 1980). Particularly noteworthy was the apparent link between repeated interpersonal experiences and diminished agency: GMAs were described not only as producing distress, but also as undermining confidence in one’s competence and willingness to express oneself. This suggests a potential pathway through which subtle interpersonal experiences may become internalized and shape subsequent workplace behavior. Although some participants reported positive effects, these accounts are better understood as context-dependent responses rather than beneficial effects of GMAs per se (Holder et al., 2015; Lilienfeld, 2017).

At the work-related level, GMAs extended beyond individual well-being to relationships and work activities. Reduced motivation and engagement co-occurred with discomfort, impaired cooperation, weakened group cohesion, communication breakdowns, and difficulties maintaining work quality and performance. These patterns are consistent with research showing that interpersonal mistreatment can deplete psychological resources and interfere with social and task-related functioning (Hobfoll, 1989; Vossoughi et al., 2025). The relational dimension appears particularly important: when trust, communication, and cooperation deteriorate, the consequences of a microaggression may extend beyond the original target–perpetrator relationship.

At the organizational level, effects were less frequently reported but nevertheless extended to organizational culture, talent development and retention, knowledge exchange, and reputation. These accounts are compatible with research linking psychologically unsafe interpersonal environments to reduced learning, knowledge sharing, innovation, and organizational functioning (Edmondson, 1999; Santana et al., 2025). Given the limited number of organizational-level accounts, however, these findings should be understood as perceived pathways of organizational impact, rather than evidence of direct organizational effects.

Taken together, the findings suggest that the significance of GMAs lies not only in individual acts, but also in their capacity to accumulate and propagate across levels of workplace functioning. Participants’ accounts suggest a perceived accumulation of consequences across levels of workplace functioning. In these accounts, subtle interpersonal behaviors were described as being associated with psychological distress, which could in turn be linked to diminished agency and engagement, while deteriorating interpersonal dynamics were perceived as having implications for cooperation and organizational functioning. This multi-level pattern offers an integrated understanding of GMAs as processes whose consequences may extend beyond the original interaction, while remaining grounded in workers’ own perceptions and experiences.

### 4.3. Reactions to GMAs in the Workplace

The findings addressing RQ3 suggest that reactions to GMAs are not simply alternative coping strategies but may form part of the process through which microaggressive experiences are recognized and acted upon.

Non-reaction was common: among the 42 participants reporting personal experiences, 29 described situations in which individuals did not respond directly. Importantly, this was often associated with shock, ambiguity, uncertainty, or perceived lack of agency rather than passivity per se (Sue & Spanierman, 2020; Sue et al., 2007). The subtle and deniable nature of GMAs may therefore constrain not only the ability to respond, but also the immediate recognition that a response is warranted.

A particularly relevant pattern in participants’ accounts was the emergence of delayed responses following repeated exposure. Several participants described individuals who initially tolerated, ignored, or were unable to respond to an incident, but who later reacted after similar episodes had accumulated. Although the cross-sectional and retrospective design does not allow this sequence to be established causally, the accounts suggest that repetition may transform an initially ambiguous event into a recognizable pattern, potentially lowering the threshold for action (Sue & Spanierman, 2020; Sue et al., 2007). In this sense, participants’ accounts suggest that cumulative exposure may shape not only the consequences attributed to GMAs, but also how targets interpret and respond to them.

Responses nevertheless remained strongly context-dependent. Participants linked the possibility and intensity of responding to personal resources, socio-economic position, and organizational circumstances, while greater seniority or security was sometimes associated with a greater perceived capacity to act (Cortina & Magley, 2003; Pearson & Porath, 2005). This is consistent with research showing that power and organizational position influence responses to workplace mistreatment (Cortina & Magley, 2009). Thus, the findings do not support a simple distinction between passive and assertive targets; rather, they point to a dynamic relationship between recognition, cumulative exposure, and perceived agency.

Overall, the most relevant insight from these accounts is that responding to a GMA may itself be an evolving process. Participants described how an ambiguous or “stunning” interpersonal event (Pierce, 1970; Williams et al., 2021) could, following repeated experiences and changes in perceived agency, become more recognizable and actionable. This interpretation extends the cumulative perspective developed above by suggesting, on the basis of participants’ accounts, that repeated experiences may influence not only perceived well-being and workplace functioning, but also how subsequent incidents are interpreted and confronted.

### 4.4. Theoretical and Practical Implications

The present study contributes to the literature by emphasizing the situated and context-dependent nature of GMAs. Rather than treating microaggressions as a uniform set of behaviors, the findings suggest that their meaning and consequences depend on gendered expectations, interpersonal relationships, power relations, and organizational context.

Accordingly, GMAs may require a gender-sensitive and context-sensitive approach to both their identification and management. Their interpretation should consider not only the behavior itself, but also the message conveyed, the gendered relationship between those involved, and the circumstances in which the interaction occurs.

The findings further suggest that these factors may also shape how individuals respond to microaggressions: a lack of overt reaction should not necessarily be interpreted as an absence of perceived offense or impact. This is particularly relevant when considering women, who represented the predominant targets in our data, while men and other gender groups should also be considered as potentially exposed to GMAs that may involve different gendered dynamics.

The results also indicate directions for future research on measurement. Existing instruments have predominantly focused on specific target groups, particularly women (e.g., Algner & Lorenz, 2022). While this focus reflects their greater exposure, measures should also capture potentially different manifestations across gender groups. Simply replacing “women” with a generic “gender” category may obscure differences in gendered expectations and experiences. Future research should therefore develop gender-sensitive instruments that allow comparison across groups while preserving group-specific manifestations and meanings. Measurement invariance across gender groups, together with observational or longitudinal approaches, could further strengthen the assessment of GMAs.

The practical implications extend beyond interventions directed at individual targets. Because the reported consequences of GMAs may accumulate across interpersonal, team, and organizational levels, prevention should not focus exclusively on those most frequently targeted. Organizational strategies should combine individual awareness with system-level prevention, including clear behavioral standards, confidential reporting mechanisms, leadership practices that reduce perceived power asymmetries, and initiatives that strengthen psychological safety and respectful workplace climates.

Finally, organizational assessment should adopt a participatory and context-sensitive approach. Employee surveys, listening initiatives, and periodic climate assessments could help identify locally salient manifestations of GMAs, barriers to reporting, and credible forms of support. Such assessments should include different gender groups without assuming equivalent experiences. In this sense, organizational resilience may depend not only on reducing individual incidents, but also on preventing subtle gendered behaviors from accumulating into broader relational and organizational dysfunction.

### 4.5. Limitations and Future Research Directions

Several limitations should be acknowledged. First, the qualitative design and non-probability sample prioritize the breadth and richness of workers’ representations over statistical representativeness and broad generalizability. The open-ended interview approach intentionally minimized predefined assumptions regarding the directionality, manifestations, effects, and reactions associated with GMAs. While this allowed participants to spontaneously identify a broad range of phenomena, it limited the possibility of examining specific patterns in depth. Moreover, the study included only cisgender women and cisgender men and therefore does not capture the experiences of transgender, non-binary, or other gender-diverse workers. Future research should examine these experiences more systematically, alongside intra-gender microaggressions, including women-to-women and men-to-men dynamics, and less explored forms of gender stereotyping.

The Italian context should also be considered when assessing the transferability of the findings. Participants’ accounts are situated within the cultural, social, and organizational norms of the Italian workplace, including context-specific understandings of gender roles, interpersonal relations, and organizational hierarchies. The study therefore does not assume that the frequency, manifestations, or meanings of GMAs would be equivalent across countries or organizational settings. Nevertheless, some of the underlying processes identified—such as the role of gendered expectations, power relations, cumulative exposure, perceived agency, and organizational climate—may also be relevant in other contexts, particularly where comparable gender norms and organizational structures are present. Their applicability should, however, be assessed empirically, considering differences in social norms, employment contexts, institutional arrangements, and gender relations. The findings are therefore best understood as contextually situated insights that may inform, rather than directly generalize to, research on workplace GMAs in other settings.

Second, the findings reflect participants’ subjective perceptions and retrospective accounts rather than independently verified workplace behaviors. The subjective nature of these accounts should not, however, be understood solely as a methodological bias, as the interpretation of the message conveyed by an interaction represents an important dimension of microaggressions. Nevertheless, retrospective accounts may be affected by recall and interpretation biases, and what participants identified as a GMA cannot necessarily be assumed to constitute behavior that would be independently classified as microaggressive. This is consistent with the exploratory aim of the study, which focused on workers’ spontaneous representations of the phenomenon.

A related limitation is that participants were not explicitly asked to distinguish between GMAs they had experienced, enacted, observed, or heard about. Although this non-directive approach allowed greater freedom in describing the phenomena associated with GMAs, it does not permit a systematic comparison of targets’ and perpetrators’ perspectives and may have favored accounts involving participants as targets, potentially due to social-desirability concerns. Future research should therefore complement such accounts with workplace observations, longitudinal designs, or other forms of methodological triangulation, as well as targeted approaches capable of eliciting perpetrators’ perspectives and examining their awareness or lack of awareness of potentially microaggressive behavior.

A further limitation is that workplace gender composition was not systematically assessed. Future research should consider the gender composition of workplaces and teams when examining GMAs, alongside broader organizational and occupational contexts, to assess how these characteristics may shape the occurrence, interpretation, and directionality of gender microaggressions.

Finally, potential interviewer effects should be considered. Although interviews were initially distributed approximately equally across male and female interviewers, the final distribution became uneven because of interviewer availability. Given the variation observed across interviewer-gender groups and the relatively young age of both interviewers, interviewer gender and age may have influenced participants’ accounts, particularly in interactions with older participants. These patterns were therefore not interpreted as independent evidence of gender differences. The over-representation of healthcare professionals (13 of 47 participants) and the limited representation of other sectors also constrain transferability across occupational contexts. Future studies using more balanced interviewer designs, broader occupational samples, and comparative or mixed-method approaches could further assess the robustness and contextual specificity of these findings.

## Figures and Tables

**Figure 1:**
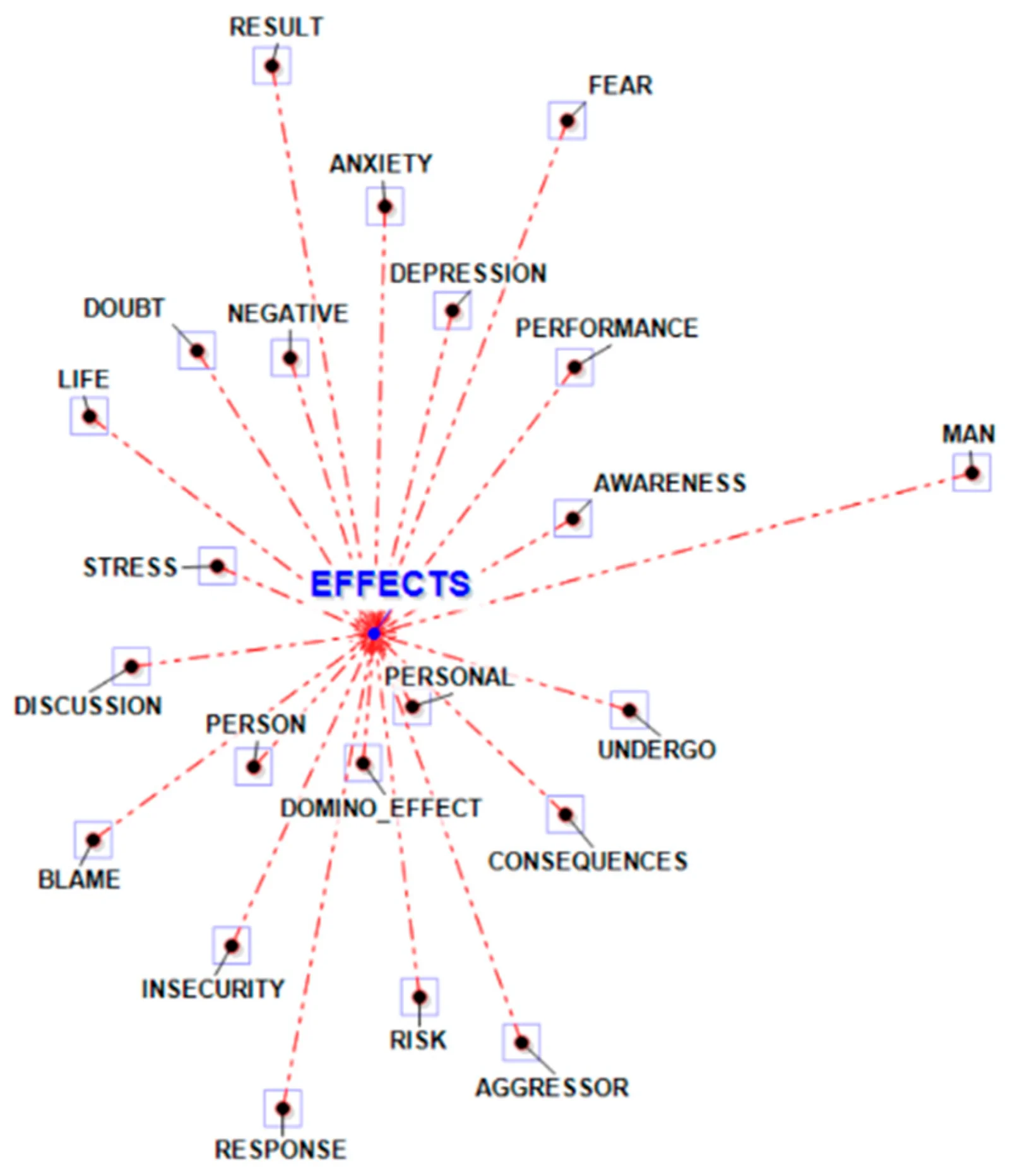
EFFECTS co-occurrence from T-Lab.

**Figure 2:**
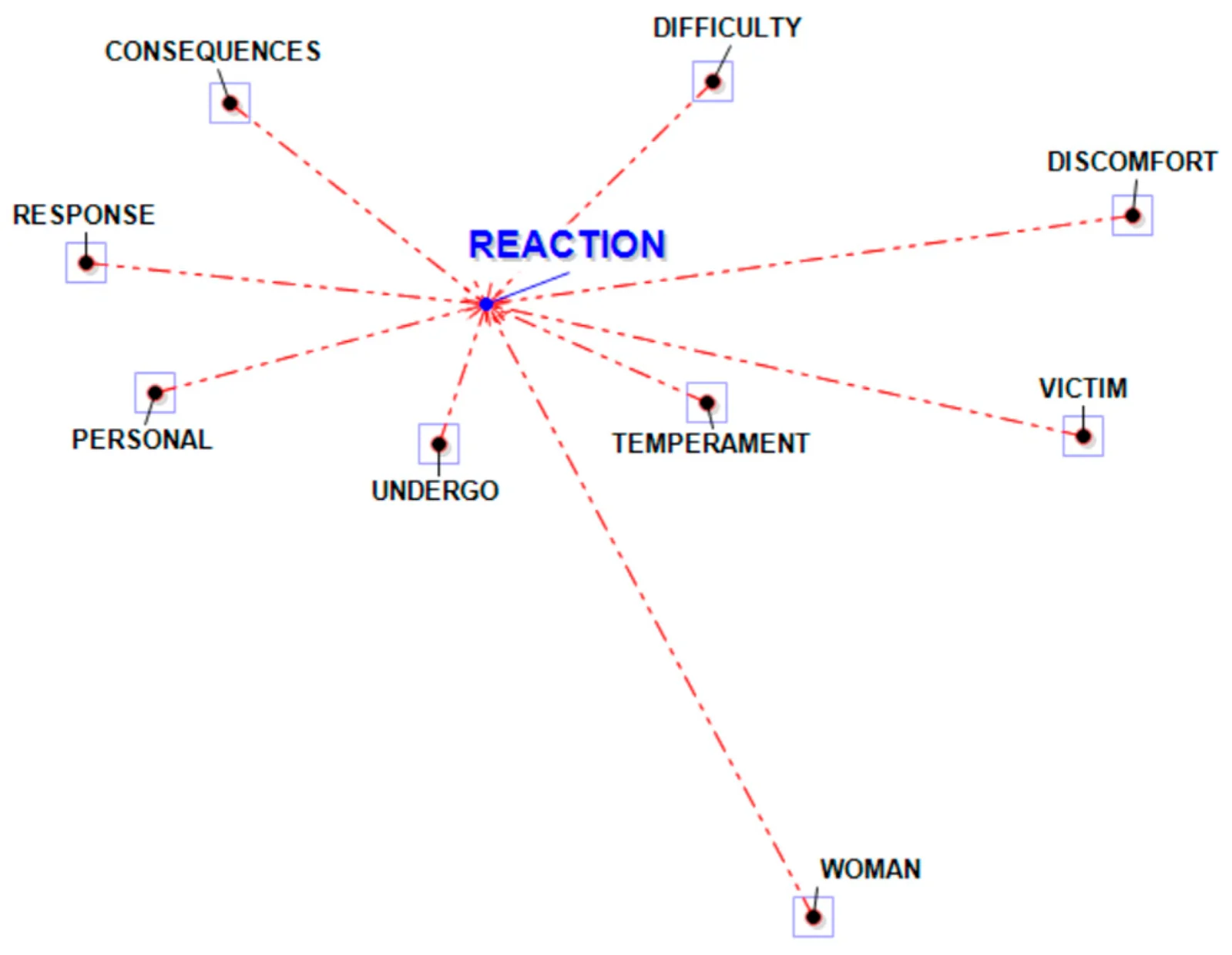
REACTION co-occurrence from T-Lab.

**Table 1:** Sample distribution by interviewer’s self-reported gender, interviewee’s gender, and age group.

Interviewers’ Gender	Interviewees’ Gender	Age		
		Under 31	31–50	Over 50
Male	Male	4	4	3
	Female	6	9	4
Female	Male	1	3	3
	Female	3	2	5

**Table 2:** Sample distribution by occupational sector.

Occupational Sector	Numerosity
Healthcare	13
HR	6
Marketing	5
Counseling	5
Legal and policy	4
Engineering	3
Administration	3
Finance and banking	2
Education	2
Other sectors	12

**Table 3:** Summary of the main thematic domains and key themes identified through the qualitative analysis.

Main Thematic Domain	Key Themes
GMA characterization and gendered directionality	Women as primary targets. Other gender minorities and men as less frequently reported targets. Cross-gender and same-gender GMAs. Perceived asymmetries in directionality.
General patterns of GMA effects	Context-dependent effects. Cumulative effects. Cascading effects across personal, interpersonal, and organizational levels.
Personal effects	Emotional and psychological distress. Self-doubt, internalization, and reduced self-esteem and self-confidence. Somatic and behavioral effects. Work–life spillover. Perceived positive outcomes (resilience and motivation).
Work-related effects	Workplace discomfort. Reduced motivation and engagement. Impaired interpersonal relationships and cooperation. Devaluation and lack of respect. Restricted freedom of expression. Defensive and submissive behaviors. Career consequences. Impaired work quality and performance.
Organizational effects	Loss of organizational capital. Reputational damage. Deterioration of organizational culture. Reduced talent development and retention.
Reactions to GMAs	Non-reaction and withdrawal. Initial shock. Context-dependent responses. Active and assertive responses. Delayed reactions.

**Table 4:** Sample distribution by interviewees’ gender, gender of microaggression targets, and directionality of gender microaggressions.

Interviewees’ Gender	Target of GMA				Directionality of GMAs			
	Women	Men	Other Categories	Homosexual Individuals	From Men Towards Women	From Women Towards Men	From Men Towards Men	From Women Towards Women
Male	18	14	6	4	14	6	6	5
Female	29	12	7	5	24	5	3	14

## Data Availability

The data presented in this study are not publicly available due to privacy and ethical restrictions. Additional information may be obtained from the corresponding author upon reasonable request and with appropriate justification.

## Supplementary Materials

The following supporting information can be downloaded at: https://www.mdpi.com/article/10.3390/bs16101864/s1, Supplementary Material S1: Codebook of GMAs’ Effects and Reactions.
