# Supplementary material for: Invisible but Impactful: Understanding the Dynamics and Effects of Gender Microaggressions Through Workers’ Eyes
Source: Behav Sci (Basel). 2026 Oct 9;16(10):1864. doi: 10.3390/bs16101864 (PMC13701453; doi:10.3390/bs16101864)
Supplement: Supplementary file 1 [file behavsci-16-01864-s001.zip › behavsci-4487330-supplementary.pdf]

Supplementary Material S1

Codebook of GMAs' Effects and Reactions

Domain	***Code***
Cross-Domain	*Context-dependence of GMAs*
Cross-Domain	*Negative chain of effects*
Personal	*Addictive behaviors*
Personal	*Anger*
Personal	*Anguish*
Personal	*Anxiety*
Personal	*Appetite changes*
Personal	*Burnout*
Personal	*Depression*
Personal	*Dissatisfaction*
Personal	*Distress*
Personal	*Embarrassment*
Personal	*Emotional effects*
Personal	*Emotional instability*
Personal	*Fear*
Personal	*Feeling personally affected*
Personal	*Frustration*
Personal	*Increased caffeine consumption*
Personal	*Loneliness*
Personal	*Mental health*
Personal	*Mood changes*
Personal	*Mood inhibition*
Personal	*Personal insecurity*
Personal	*Positive effects*
Personal	*Pride/Self-confidence/Self-esteem*
Personal	*Rumination*
Personal	*Self-awareness*
Personal	*Self-conviction*
Personal	*Self-doubt*
Personal	*Self-fulfillment*
Personal	*Sense of injustice*
Personal	*Serenity*
Personal	*Sexual desire*
Personal	*Shame*
Personal	*Somatization*
Personal	*Spillover into private life*
Personal	*Strategic Victimhood*
Personal	*Stress*

Personal	*Suppressing negative emotions to avoid affecting others in private life*
Personal	*Unhappiness*
Personal	*Victim well-being*
Personal	*Withdrawal*
Personal	*Work–life balance*
Work-related	*Aggressive behaviors*
Work-related	*Aggressor's reputation*
Work-related	*Being dismissed/Terminated*
Work-related	*Being ignored/Belittled*
Work-related	*Career advancement*
Work-related	*Communication problems*
Work-related	*Defensive behaviors*
Work-related	*Devaluation*
Work-related	*Diminishment*
Work-related	*Empathy for the victim*
Work-related	*Engagement*
Work-related	*Freedom of expression*
Work-related	*Freedom to express oneself/Work as desired*
Work-related	*Group cohesion*
Work-related	*Guilt for not being the victim*
Work-related	*Increased effort to avoid further negative consequences*
Work-related	*Inequality/Preferential treatment*
Work-related	*Interpersonal conflicts*
Work-related	*Interpersonal relationships*
Work-related	*Isolation*
Work-related	*Job dissatisfaction*
Work-related	*Mental clarity*
Work-related	*Motivation*
Work-related	*Negative workplace climate*
Work-related	*Organizational commitment*
Work-related	*Overall work-related well-being*
Work-related	*Perceived need to do more*
Work-related	*Perceived ownership/Control*
Work-related	*Powerlessness/Giving up*
Work-related	*Proactivity*
Work-related	*Quitting one's job*
Work-related	*Recognition*
Work-related	*Resignation*
Work-related	*Respect*
Work-related	*Sense of inadequacy*
Work-related	*Silence/Submission*
Work-related	*Submissive behavior*

Work-related	*Suppression of diversity*
Work-related	*Teamwork/Cooperation*
Work-related	*Uncrossable boundaries between*
Work-related	*Work identification*
Work-related	*Work organization*
Work-related	*Work quality/Performance*
Work-related	*Workplace discomfort*
Work-related	*Work-related errors*
Organizational	*Absenteeism*
Organizational	*Authoritarian climate*
Organizational	*Economic consequences*
Organizational	*Mobbing*
Organizational	*Organizational capital/Knowledge exchange*
Organizational	*Organizational image/Reputation*
Organizational	*Organizational issues*
Organizational	*Organizational stagnation*
Organizational	*Talent development and retention*
Organizational	*Toxic organizational culture*
Organizational	*Turnover*
Reaction	*Active response*
Reaction	*Concealed indignation behind a facade*
Reaction	*Context-dependent reaction*
Reaction	*De-escalating Response*
Reaction	*Delayed response after repeated exposure*
Reaction	*Difficulty in responding assertively*
Reaction	*Initial shock and disorientation*
Reaction	*Non-reaction*
Reaction	*Physical expression of reaction*
Reaction	*Private emotional venting*
Reaction	*Withdrawal from the situation*
Reaction	*Would now react differently*
